# Detection of Gravitational Wave Emission by Supermassive Black Hole Binaries Through Tidal Disruption Flares

**DOI:** 10.1038/srep35629

**Published:** 2016-10-21

**Authors:** Kimitake Hayasaki, Abraham Loeb

**Affiliations:** 1 Department of Astronomy and Space Science, Chungbuk National University, Cheongju 361-763, Korea; 2 Harvard-Smithsonian Center for Astrophysics, 60 Garden Street, Cambridge, MA 02138, USA

## Abstract

Galaxy mergers produce supermassive black hole binaries, which emit gravitational waves prior to their coalescence. We perform three-dimensional hydrodynamic simulations to study the tidal disruption of stars by such a binary in the final centuries of its life. We find that the gas stream of the stellar debris moves chaotically in the binary potential and forms accretion disks around both black holes. The accretion light curve is modulated over the binary orbital period owing to relativistic beaming. This periodic signal allows to detect the decay of the binary orbit due to gravitational wave emission by observing two tidal disruption events that are separated by more than a decade.

Most galaxies are inferred to harbor supermassive black holes (SMBHs) with masses in the range of 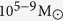 at their centers^1^,^2^, based on observations of stellar proper motion^3^, stellar velocity dispersion^4^ or accretion luminosity^5^. Most of time, their nuclei do not exhibit significant activities^6^, but in a small fraction of all galaxies the inflow of cold gas triggers active galactic nuclei (AGNs) which produce intense radiation and powerful outflows. Tidal disruption events (TDEs) provide a distinct opportunity to probe dormant SMBHs in inactive galaxies (see a recent general review^7^).

SMBH binaries with a sub-parsec (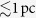) separation are considered to be the end product of a galaxy merger before two black holes finally coalesce^8^,^9^. The merger of two SMBHs goes through the following processes^10^: first, the SMBHs sink towards each other via dynamical friction on the surrounding stars and gas. Once their separation drops below a parsec, a hard binary forms^11^ and tightens further due to other mechanisms^12^,^13^ until gravitational wave emission takes over at the final stage^14^.

The observed TDE rate on single SMBHs is ~10^−5^ yr^−1^ per galaxy^15^,^16^, although another recent survey suggests that the rate could be one-order of magnitude higher^17^. This higher rate has been implied by the recent theoretical works based on two-body relaxation^18^,^19^. However, SMBH binaries are expected to exhibit an enhanced TDE rate of up to 10^−1^ yr^−1^ from chaotic orbital evolution and the Kozai-Lidov effect^20^,^21^,^22^. Recent work suggests that multiple TDEs may occur in merging SMBH binaries with a rate of up to a few times over an observing period of five years with the *Large Synoptic Survey Telescope* (LSST, http://www.lsst.org)^23^. It is less clear what characteristic flares such TDEs would exhibit, although interruptions in the light curve are likely if the orbital period of SMBH binaries is less than the period of an observable emission^24^,^25^,^26^.

If the binding energy of the approaching star relative to either component of the binary is negative inside the tidal disruption radius, the star should be finally tidally disrupted. Here, we study how stars migrating through the binary Lagrange points are tidally disrupted by the SMBHs. In Section 2, we describe the numerical method used in our simulations of these TDEs. We then analyze our numerical results in Section 3. Finally, we summarize our main conclusions in Section 4.

## Methods

### Test-particle limit

We consider a system composed of a star orbiting around a circular SMBH binary with mass ratio *q* = *M*_2_/*M*_1_ = 0.1. The initial position and velocity in our simulation are correspondingly the Lagrange point L2 and the Keplerian velocity there. This situation would be, for example, realized in the case of a massive circumbinary disk^27^, where the disk makes stars migrate inwards and approach the Lagrange points around the binary (L2 or L3) via a mass stream from the disk’s inner edge^28^. The dynamics of the star can be treated as a restricted three-body problem. We solve numerically the motion of the star as a test particle in the binary potential.

**Figure 1:**
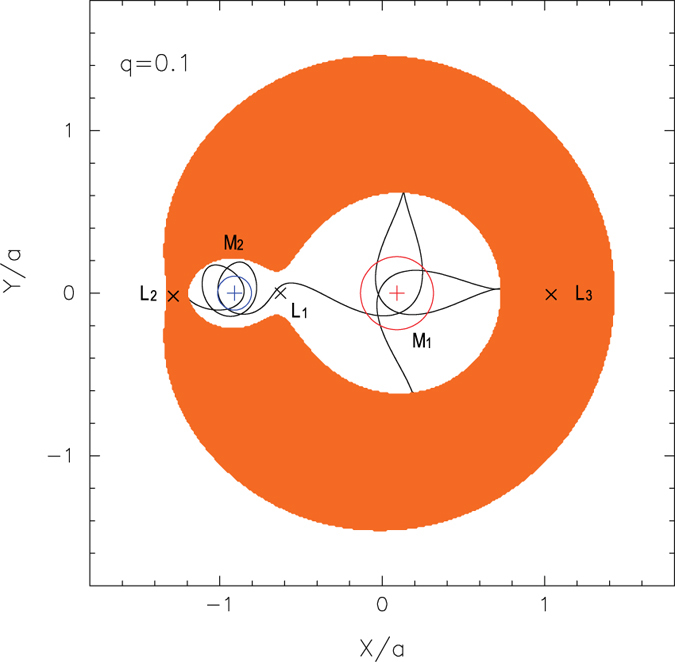
Orbit of a test particle in a co-rotating frame around a circular binary with *M*_2_/*M*_1_ = 0.1. The red and blue circles shows the tidal disruption radii around the primary and secondary black holes, respectively. The three black crosses mark the Lagrange points, *L*_1_, *L*_2_, and *L*_3_. Both axes are normalized by the binary semi-major axis. The black solid line shows the motion of a test particle inside the zero-velocity curve for a Jacobi constant *C*_J_ = 3.45, and the shaded area outside the curve denotes the forbidden zone^29^.

In the test-particle limit, the star is initially located just inside the L2 point with a Keplerian velocity, and then is trapped by the binary potential and freely moves between two black holes inside the inner Roche-lobe of the binary. The outside of the inner lobe is marked by the shaded area of Fig. 1 as the forbidden zone^29^. The star is tidally disrupted once it enters the tidal disruption radius:


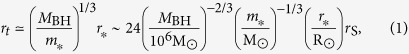


where *M*_BH_ is the SMBH mass, m_*_ and r_*_ are the star’s mass and radius, and *r*_S_ = 2G*M*_BH_/c^2^ is the Schwarzschild radius. The corresponding orbit is depicted in Fig. 1. The fate of the tidally disrupted star is similar to that of an eccentric TDE^30^, in which the TDE accretion rate deviates significantly from the standard *t*^−5/3^ time evolution^31^,^32^.

### SPH simulations

Our simulations are performed with a three-dimensional Smoothed Particle Hydrodynamics (SPH) code^33^. The hydrodynamic equations are integrated using a second-order Runge-Kutta-Fehlberg integrator with individual time steps for each particle, leading to substantial savings in time for a large dynamic range of timescales. We also use a variable smoothing length scheme to find the relevant spatial resolution in our code, but ignore the term proportional to the gradient of the smoothing length. We adopt standard the SPH artificial viscosity parameters *α*_SPH_ = 1 and *β*_SPH_ = 2.

The photon diffusion timescale of the stellar debris is given by


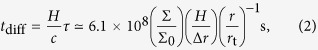


where *H* is the debris scale height and *τ* = *κ*_es_Σ ~ 2.6 × 10^6^ (Σ/Σ_0_) is the optical depth for electron scattering with *κ*_es_ = 0.4 cm^2^ g^−1^, and Σ is surface density of the stellar debris with the fiducial value of


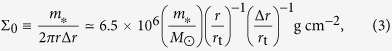


where *r* and Δ*r* are the radius and width of the debris ring. Recent three-dimensional radiation magneto-hydrodynamic simulations showed that the magnetic buoyancy induced through the magneto-rotational instability (MRI)^34^ provides an efficient radiative cooling mechanism for super-Eddington accretion flows^35^. In this regime, their preliminary simulations indicate that the radiative cooling speed is determined by the sound speed of the radiation. In our simulation, the stellar debris loses its orbital energy and then circularizes via a shock into an accretion flow at a super-Eddington rate at ~*r*_t_. The initial radiation energy density is 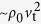, where *ρ*_0_ = Σ_0_/*r*_t_ and 
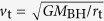
, and so the radiation sound speed is estimated to be 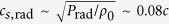. Thus, the resulting cooling timescale *r*_t_/*c*_s,rad_ is estimated to be ~2.8 × 10^3^ s, shorter than the heating timescale by the shock 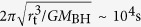. It takes several dynamical times for the magnetic field to grow via the MRI, after which the efficient cooling reduces the thickness of the debris streams. Thus, we use a simple polytropic equation of state: *P* = *Kρ*^*γ*^, where *P* and *ρ* are the gas pressure and density, *γ* = 5/3 and *K* is a constant. This ensures that the radiative cooling is efficient throughout our simulation.

We model the initial star as a polytrope with the polytrope index *n* = 1.5 in hydrostatic equilibrium. The star is set in motion through the gravitational field of the SMBH binary with the following parameters: 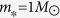, 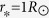, 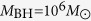, *a* = 100*r*_S_, and *q* = 0.1. The simulations follow 0.5 million SPH particles for 14 binary orbital periods, where a single period *P*_orb_ ~ 1 day.

## Results

### Tidal disruption of a star

**Figure 2:**
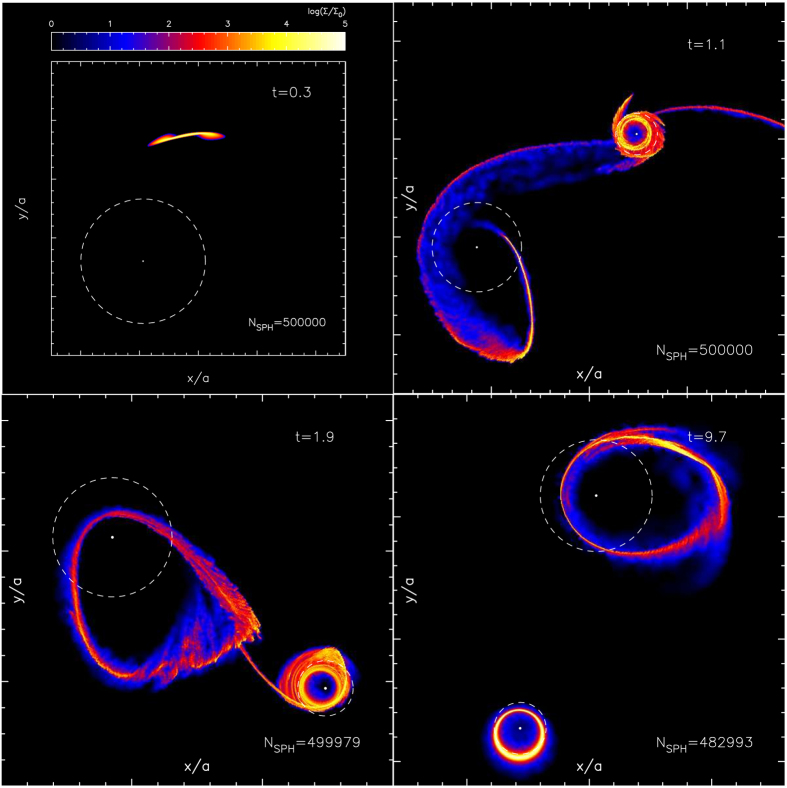
Gas density maps, projected on the orbital (x-y) plane, during a tidal disruption event in a circular SMBH binary with 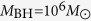, *a* = 100*r*_S_, and *q* = 0.1. The run time *t* is in units of *P*_orb_ as labeled in the top-right corner of each panel. The number of SPH particles is indicated at the bottom-right corner. The panels are shown in chronological order. The coloration indicates the density in five orders of magnitude on a logarithmic scale normalized by Σ_0_ = 6.5 × 10^6^ g cm^−2^. The dashed circles indicate the tidal disruption radii for the primary and secondary SMBHs.

Figure 2 shows the evolutionary sequence of density maps for the stellar debris of a TDE around the SMBH binary. A star approaching the binary from the L2 point is tidally disrupted by the secondary black hole. Part of the stellar debris is not gravitationally bound, while the rest accretes onto the secondary SMBH. The unbound debris moves chaotically in the binary potential and forms accretion disks around both black holes by transferring mass along the potential valley through the L1 point.

**Figure 3:**
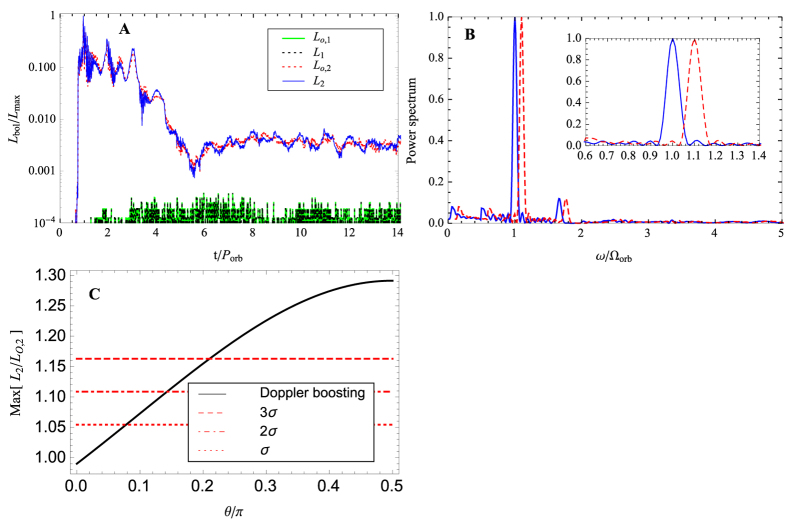
(**A**) Bolometric luminosity of the primary (black and green) and secondary (red and blue) black holes normalized by its maximum value *L*_max_ = max{*L*_o,1_ + *L*_o,2_}. The black and blue lines include relativistic beaming. (**B**) Power Spectrum of the bolometric light curves with relativistic beaming as a function of frequency *ω* in units of Ω_orb_ = 2*π*/*P*_orb_. The blue solid line shows the power spectrum of the first TDE, and the red dashed line shows that of the second TDE 50 years later. The half maximum full-width are given by 0.05240 ± 0.000563 with a fixed peak position 0.99027. If the two peaks are sampled by *N* data points, it should be possible to separate them even after a time interval of 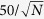 years. (**C**) Inclination angle dependence of the maximum value of *L*_2_/*L*_*o*,2_. The dashed, dot-dashed, dotted lines are the ones corresponding to 1 to 3 *σ* of the fluctuations in *L*_*o*,2_.

The resulting mass accretion rates are shown in Fig. 3A. The green and red dotted lines show the bolometric light curves of the primary and secondary black holes, respectively. The luminosities are derived as 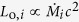 with *i* = 1, 2, where 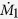 and 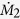 are the mass accretion rates of the primary and secondary black holes based on the simulation. The accretion rates are estimated at the radius of the innermost stable circular orbit of a Schwarzschild black hole, 3*r*_S_. The black and blue lines show the bolometric light curves, including the relativistic doppler beaming effect due to the orbital motion of the binary^36^,


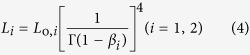


where 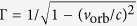 and 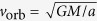 are the Lorentz factor and the orbital speed, respectively, and


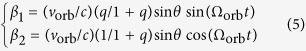


with 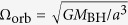 being the orbital frequency and *θ* being the inclination angle between the line-of-sight and the binary orbital plane, which is assumed to be *π*/2 in the current simulation. Note that 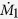 intrinsically shows the bursty nature and is more than one order of magnitude smaller than 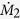, since the angular momentum of the stellar debris relative to the primary black hole is much larger than that of the secondary black hole. In addition, the periodicity of the bolometric light curves of the secondary black hole is enhanced by relativistic beaming.

Figure 3B shows the power spectrum of the total light curve with relativistic beaming. The blue solid and red dashed lines mark the simulated and predicted power spectra, respectively. The blue line indicates a sharp peak at the binary orbital frequency. Since the full-width at half maximum of the peak is ~0.052Ω_orb_, the shift of the peak over time due to orbital decay should be larger than ±0.052Ω_orb_ in order to be easily noticeable. For a fiducial TDE rate of ~0.02 per year, a second TDE would likely occur 50 years after the first TDE, and the shift in the power spectrum due to the orbital decay by gravitational wave emission will be noticeable, as shown by the red dashed line of the figure. If the peak is sampled by N data points, then the shift in the peak would be detectable over a period shorter by 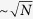. Our simulation implies that it would be possible to measure the orbital decay by gravitational wave emission through the shift of the peak in the power spectrum.

Figure 3C shows the dependence on the inclination angle, *θ*, of the amplitude of relativistic doppler boosting relative to the amplitude of the original bolometric light variation emitted from the secondary black hole. The black line represents *L*_2_/*L*_*o*,2_ (see equation 4), whereas the dashed, dash-dotted, and dotted lines represent the amplitudes corresponding to 3*σ*, 2*σ*, and 1*σ* of the fluctuating *L*_*o*,2_, respectively. The panel indicates that the periodic signal corresponds to 3*σ* detection if 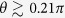.

### Shift in the binary orbital period

The feasibility of detecting the shift in the peak of the power spectrum depends primarily on the semi-major axis of the binary at the first TDE. We consider a circular binary whose coalescence timescale due to gravitational wave emission is given by^37^,


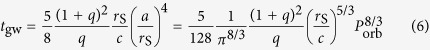


Setting *a*_0_ and *a* as the semi-major axes of the binary at the first and second TDEs, respectively, the time difference between two TDEs, Δ*t*_gw_, needs to satisfy the condition that the normalized frequency is significantly increased, namely


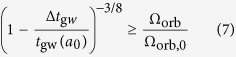


Thus, the upper limit on a value of semi-major axis *a*_0_ is,


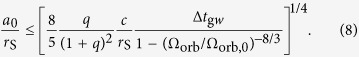


**Figure 4:**
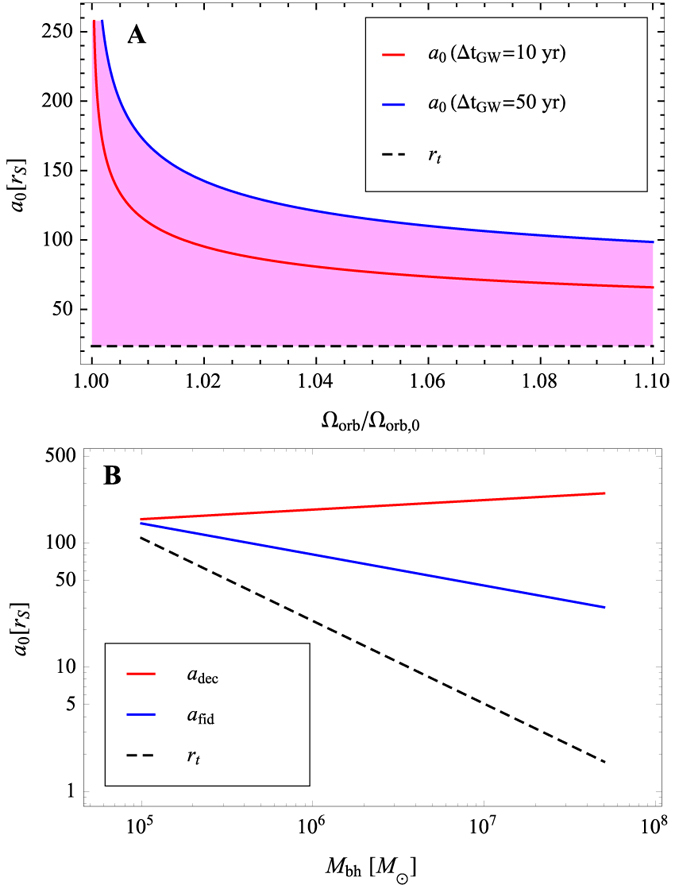
(**A**) Dependence of the semi-major axis at the first TDE, *a*_0_, on the orbital frequency at the second TDE, normalized by the orbital frequency at the first TDE, for 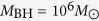. The blue and red solid lines show *a*_0_ if the time difference between the first and second TDEs is given by 10 and 50 years, respectively (see equation 8). The black dashed line shows the tidal disruption radius *r*_t_ below which the binary can be regarded as a single black hole (see equation 1). (**B**) Dependence of the decoupling radius on black hole mass. The red, blue and black dashed lines show the decoupling radius, fiducial semi-major axis, and tidal disruption radius, respectively.

Figure 4A shows the dependence of *a*_0_ on Ω_orb_/Ω_orb,0_. The blue and red solid lines denote *a*_0_ if Δ*t*_g*w*_ is given by 10 and 50 years, respectively. The black dashed line shows the tidal disruption radius *r*_t_ below which the binary can be regarded as a single black hole (see equation 1). The range of the semi-major axis at the first TDE, which is detectable, is shown by the shaded area.

## Summary and Discussion

We have simulated the tidal disruption of a star by a circular SMBH binary with a 1:10 mass ratio. Our simulation indicates that the mass accretion rate of the secondary black hole is higher than that of the primary black hole. The resulting total bolometric luminosity shows a strong relativistic beaming effect due to the enhanced line-of-sight velocity of the secondary black hole. Therefore, the power spectrum naturally shows a sharp peak at the orbital frequency (see Fig. 3B).

### Interaction with a circumbinary disk

If the SMBH binaries are located in a gas-rich environment, a circumbinary disk can be the agent supplying the stars. Accretion disks around SMBHs are predicted to be gravitationally unstable at large radii where they fragment into stars or planets owing to their self-gravity^38^,^39^,^40^. Similarly, a circumbinary disk could form stars in its outskirts. Hence, a self-gravitating circumbinary disk would naturally lead to be an enhanced TDE rate^27^. In addition, a circumbinary disk could grind the orbits of background stars and trigger additional TDEs^41^,^42^. Through both mechanisms, stars would migrate to the inner edge of the circumbinary disk, and subsequently approach both black holes through the Lagrange points (L2 or L3) of the binary.

When *t*_gw_ is shorter than the viscous timescale evaluated at the inner edge of the circumbinary disk, the binary is viscously decoupled from the circumbinary disk^43^. The viscous timescale measured at the inner edge of the circumbinary disk is given by *t*_vis_ = *r*^2^/*ν* with the inner radius being ~2*a*^44^, where the disk viscosity *ν* = *α*_SS_*c*_s_*H*, expressed in terms of the sound speed *c*_s_ and the Shakura-Sunyaev viscosity parameter *α*_SS_. Based on the assumption that the circumbinary disk is a standard disk, the decoupling radius is obtained by equating *t*_vis_ to *t*_gw_, giving


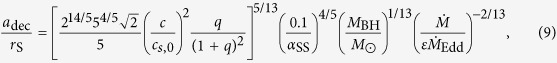


where *c*_s,0_ ~ 1.7 × 10^8^ cm s^−1^ is the sound speed for typical parameters. Figure 4B shows the black-hole mass dependence of the decoupling radius. The decoupling radius is clearly larger than the fiducial semi-major axis *a*_fid_ = 100*r*_S_ of our simulation for 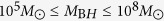.

Recent numerical simulations show that the rate of gas accretion from the circumbinary disk is modulated by the orbital period^45^,^46^. Detection of the orbital decay based on the shift in the power spectrum of the light curves can also, in principle, be inferred in such a system. However, if the semi-major axis of the binary is larger than the decoupling radius, the shift of power spectrum is undetectable because it is too small to be identified observationally (Ω_orb_/Ω_orb,0_ < 1.01 in Fig. 4A). This implies that only periodic signals caused by tidal disruption events around the SMBH binaries with the semi-major axis shorter than the decoupling radius can provide a detectable shift in the power spectrum.

The TDE flares are attributed to super-Eddington accretion phases of short duration^47^,^48^. They differ from a sub-Eddington accretion mode which may last much longer, as illustrated in Fig. 3 of Farris *et al*.^49^. The surface density of the stellar debris in our simulations is many orders of magnitude higher than that of a sub-Eddington accretion flow. We therefore expect that any pre-existing accretion disk would not affect significantly the dynamics of the debris.

The TDE rate for a binary SMBH system is dictated by the supply rate of stars from the outer region of the circumbinary disk to the inner cavity. The formation rate of the solar mass stars is estimated to be of order 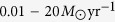 in the self-gravitating accretion disk^40^. If the stars can induce gap formation in the disk, they would migrate on a timescale that cannot be shorter than the Eddington accretion timescale as they are coupled to the viscous evolution of the disk. In this case, the expected TDE rate ranges between 0.01 yr^−1^ and the Eddington rate, 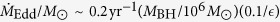, where 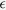 is the mass-to-radiation conversion efficiency. On the other hand, the rate at which the orbits of disk-crossing stars are brought into the inner cavity could also be controlled by the hydrodynamic drag force acting on the trapping stars^42^,^50^. In this case, stars of a solar mass should migrate to the inner cavity on the radial drift timescale 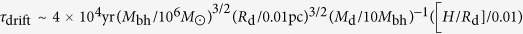 for the relatively massive, geometrically thin disk, where *R*_d_ and *H* are the disk radius and scale-height^50^. Therefore, the expected TDE rate is suppressed by the radial drift timescale down to 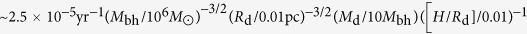.

### Detectability of the events

The light curve of the radiation emitted by the jet, whose flux is proportional to the mass accretion rate obtained in our simulation, would make it possible to distinguish a binary TDE from the usual emission in AGNs based on the following characteristics: 1. the angle between the line of sight and jet direction should change transiently for a TDE, even if there is steady feeding of the black hole. 2. the jetted emission shows clear periodicity during several tens of binary orbital periods.

LSST would be ideally suited for identifying candidate source for follow-up monitoring that will seek our predicted periodic signal. Given that a typical galaxy undergoes a merger with a mass ratio 1:10 once every ~1 Gyr^51^, the probability for finding a corresponding SMBH binary in the last century of its life before coalescence is ~(10^2^ yr/10^9^ yr) ~ 10^−7^. Since the LSST will survey 3.7 × 10^10^ galaxies for variability during its 10 year operation (http://www.lsst.org), it is expected to find 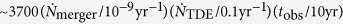 events. In order to obtain the 3 sigma detection of the periodicity by relativistic doppler boosting, we multiply the above number by the factor 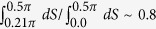, where *dS* = 2*π* sin *θdθ* is the differential solid angle for *θ* direction, from Fig. 4C. Therefore, we expect to detect 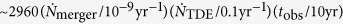 signals from the SMBH binaries with a detectable shift over a period of 10 year.

## Additional Information

**How to cite this article**: Hayasaki, K. and Loeb, A. Detection of Gravitational Wave Emission by Supermassive Black Hole Binaries Through Tidal Disruption Flares. *Sci. Rep.* **6**, 35629; doi: 10.1038/srep35629 (2016).
